# The Impact of the COVID-19 Epidemic During the Lockdown on Children With the Pediatric Acute-Onset Neuropsychiatric Syndrome (PANDAS/PANS): The Importance of Environmental Factors on Clinical Conditions

**DOI:** 10.3389/fneur.2021.702356

**Published:** 2021-08-11

**Authors:** Cristiana Alessia Guido, Lorenzo Loffredo, Anna Maria Zicari, Piero Pavone, Salvatore Savasta, Antonella Gagliano, Giulia Brindisi, Giuliana Galardini, Antonella Bertolini, Alberto Spalice

**Affiliations:** ^1^Department of Pediatrics, Child Neurology Division, Sapienza University of Rome, Rome, Italy; ^2^Department of Developmental and Social Psychology, Faculty of Medicine and Psychology, Sapienza University of Rome, Rome, Italy; ^3^Department of Clinical, Internal, Anaesthetic, and Cardiovascular Sciences, Sapienza University of Rome, Rome, Italy; ^4^Department of Pediatrics, Sapienza University of Rome, Rome, Italy; ^5^Department of Clinical and Experimental Sciences Medicine, Division of Pediatrics and Child Neuropsychiatry, University of Catania, Catania, Italy; ^6^Division of Pediatrics - Azienda Socio Sanitaria Territoriale (ASST) Crema, Crema, Italy; ^7^Child Neuropsychiatry Operating Unit, University of Cagliari, Cagliari, Italy; ^8^Italian Association PANDAS Organizzazione di Volontariato Italia ODV, Firenze, Italy

**Keywords:** PANS, PANDAS, neuropsychiatric syndrome, COVID-19, stress

## Abstract

**Introduction:** In March 2020, SARS-CoV-2 declared a pandemic by the World Health Organization. Restrictive isolation measures have also brought psychological distress to the pediatric population. Building on the syndrome's characteristics, the present study explored the impact of lockdown on the clinical course of young people with PANDAS/PANS. The initial hypothesis considered both the reduced exposure to viral agents and the strategies of the parents and other containment actions as protective factors against the worsening of symptoms.

**Methods:** One hundred and eight children, adolescents, and young adults were recruited according to the multicenter PANDAS/PANS research program. Parents participated in a web-based survey. Results: contrary to our hypothesis, the study results show an increase in symptoms during the block in 71% of the sample. Psychometric analyzes allowed us to exclude a relationship between the main symptoms of PANDAS and the increase in symptoms or the presence of symptoms before the block and their increase over time. The increase in symptoms is best explained by the presence of sleep disturbances and emotional lability. The exacerbation also appears to be linked to the onset of new symptoms in children and adolescents with depressed moods and eating problems. Furthermore, irritability and oppositionality are significant predictors of acute exacerbation. Equally statistically significant is the factor linked to the effects of pandemic stress, such as the fear of contracting the virus. No significant associations for symptom reduction have been identified between parental strategies or other parent-initiated actions, but the study demonstrates that caregiver perceived efficacy on the strategies used can reduce the risk of exacerbation.

**Conclusion:** This preliminary study highlights the importance of studying the causes of increased symptoms in children with PANDAS/PANS. Life events can exacerbate the clinical condition or generate new symptoms in young patients. In particular, environmental, family, and social changes in the course of clinical symptoms in PANDAS/PANS patients should be investigated. It highlights the importance of emotional and behavioral management, which can be improved by enhancing coping strategies in young people with PANDAS/PANS and their caregivers through a combination treatment in which CBT and PMT are included, in line with guidelines.

**Limits:** An experimental proxy-report questionnaire not yet standardized and validated on the PANS/PANDAS pediatric clinical sample was used for the exploratory study. There is also a small sample size (*N* = 108) and the absence of a control group (pre-lockdown or children without PANDAS/PANS). It would be interesting to evaluate the exact long-term dimensions to see the course of symptoms after covid and conduct a new study focusing on the impact of stressful events on the clinical course of the syndrome.

## Introduction

### The PANS and PANDAS Syndromes

The National Institutes of Health (NIH), in the 1980s, found obsessive-compulsive symptoms with sudden onset following streptococcus pyogenes, varicella, and Mycoplasma pneumoniae infections (Pediatric Infection Triggered Autoimmune Neuropsychiatric Disorders) in a group of children -PITANDS) (1, 2). Subsequent research on cohorts of children with acute-onset OCD related to group A streptococcal infections (GAS) preceded by a prodrome of Sydenham's chorea (SC) suggested that acute-onset OCD might be a consequence of SC (3, 4). Subsequent systematic studies of the relationship between sudden onset and episodic course of tics or obsessive-compulsive symptoms generated by GAS infections led to the classification of a new syndrome of “pediatric autoimmune neuropsychiatric disorders associated with streptococcal infections” called PANDAS (5). Because of the variability of symptoms and their evolution, the diagnosis remains controversial. In 2012, Swedo et al. published diagnostic criteria for PANS. An “acute and dramatic onset of symptoms” (pediatric acute-onset neuropsychiatric syndrome) must occur to clinically define PANS (6), which can occur in the pediatric population (up to 18 and, in some definitions, 21 years). Experts have also included a broad spectrum of symptoms among the diagnostic criteria potentially associated with prior infection as in PANDAS and acute neuropsychiatric disorders without apparent precipitating environmental impact or immune dysfunction. Thus, PANS must be characterized by many interrelated heterogeneous disorders and an acute (lightning) onset or must have a relapse of OCD associated with two or more neuropsychiatric symptoms (7). Severe psychotic symptoms can sometimes emerge in the clinical setting of PANS/PANDAS (8).

Furthermore, emotional lability/depression could be highly related to autoimmune diseases in pregnancy, while anxiety symptoms could be related to recurrent infectious diseases in PANS patients (9). A recent study also found a possible involvement of serum levels of NOX2, as well as 8-iso-prostaglandin F2α (8-iso-PGF2α) and lipopolysaccharide (LPS) in neuroinflammatory processes in PANDAS PANDAS patients (10). The criteria of Swedo et al. were confirmed in the first PANS Consensus Conference in 2013 (7). Within the Diagnostic and Statistical Manual of Mental Disorders DSM-fifth edition (11), the diagnostic features of PANDAS were introduced in the category “Obsessive-compulsive disorder and related disorders due to another medical condition 294.8 (F 06.8)” (…) characterized by the sudden onset of obsessions, compulsions, and/or tics accompanied by a variety of acute neuropsychiatric symptoms, in the absence of chorea, carditis, or arthritis, following a group A streptococcal infection. The DSM-5 also specifies that pending further clinical studies. Although there is some evidence to support PANDAS, this remains a controversial diagnosis. Given the ongoing controversy, in order to eliminate etiologic factors and indicate a consistent clinical entity: the description of PANDAS was modified to indicate idiopathic acute childhood neuropsychiatric syndrome (PANS) or acute neuropsychiatric symptoms (CANS). In 2017, the consensus guidelines for the treatment of children with PANS and PANDAS (1), given the symptomatic overlap between the two syndromes (obsessions and compulsions; food restriction; irritability and aggression; anxiety; ADHD symptoms; sleep disturbance; depression; and pain in the musculoskeletal area), decided to provide a treatment plan considering PANS/PANDAS as a single entity.

Regarding therapy, a combined approach is recommended by administering immunomodulatory drugs and antibiotics together with cognitive-behavioral psychotherapy (CBT) using the technique of exposure and response prevention (ERP). Additionally, Parent Management Training (PMT) can help caregivers manage children's problem behavior. The role of parents assumes great importance in clinical management, mainly due to the relapsing-remitting course of the disease. In the acute stages, emotional and behavioral symptoms can be a stressor for the patient's family. Among strategies for parents, experts recommend coordinating medical care and interacting with the school. Parents are also advised not to indulge the child's compulsive and obsessive ritual demands and to offer reassurance during times of anxiety and severe irritability. Instead, in stages of symptom remission, parents should resume their usual strategies, establish behavioral boundaries, reinforce desirable behaviors, and ignore or punish undesirable behaviors by removing privileges (1).

To the best of our knowledge, few studies have data on the efficacy of psychotherapeutic treatments because they focus primarily on viral etiology, considering it the sole source of symptom triggering in the flare-ups. Given this premise, it is essential to analyze all factors contributing to symptom exacerbation, including environmental factors and triggering events of social, familial, and educational nature.

Research initiated during the COVID-19 pandemic on normal and clinical samples showed that changes in routine produced emotional and behavioral reactions in the pediatric and adolescent population.

Coronavirus infection can also affect children who develop mainly mild symptomatic forms in which the most frequent clinical presentation, in addition to fever, may involve olfactory and gustatory loss, respiratory distress, and gastrointestinal manifestations. The SARS-CoV-2 disease is often linked to a multisystem inflammatory syndrome (MIS-C), which mimics the clinical features of Kawasaki disease (KD) (12–14). Psychophysiological symptoms appear to be predominantly associated with social and family changes adopted during the pandemic. SARS-CoV-2 spread initially in China and worldwide, has caused a pandemic recognized by the World Health Organization, also imposing to Italy, from March 2020, the closure of schools, gyms, and places of socialization to bend the curve of contagion (15).

### Psychological Condition in Children and Adolescents During the Pandemic

The effects of the COVID-19 pandemic on mental health in children and adolescents without previous problems show the onset of anxiety-depressive symptoms, stress, and somatic symptoms (body pain, breathing difficulties) (16–19); neurophysiological alterations such as dysregulation in sleep/wake rhythms and feeding, the presence of fatigue and increased inattention and irritability; and increased attachment to caregivers with demands for reassurance (20–23). In the clinical population, a significant increase in the frequency of contamination obsessions and cleaning/washing compulsions was found in samples of children with a primary diagnosis of OCD (24) while, patients with Gilles de la Tourette syndrome developed new repeated cough tics also evident in public during the pandemic (25). A longitudinal study of 141 children and adolescents with previous neurological disorders, psychiatric problems, and complex developmental problems also showed a significant worsening of somatic and anxiety symptoms in children aged 1.5 and 5 years and the presence of thinking problems, obsessive symptoms, and post-traumatic stress symptoms from ages 6 to 18 years. In addition, the group of patients with psychiatric problems showed a better response to blocking due to reduced environmental demands (26).

Among risk factors related to the onset of symptoms during the lockdown, studies have shown a link to parental stress and mental and physical problems, family tension, and lower levels of resilience in children. Changes in parental working conditions, anxiety transmitted from parents to children, and fear of contagion associated with a perceived threat to life caused by uncontrollable events may also promote increased psychological distress in children (18, 23, 27, 28). Changes in daily routine, social isolation, boredom, lack of adequate information about the virus, and limited living space are other risk factors (21, 29). In terms of gender and age-related risk in children and adolescents, studies show controversial results, which require further investigation (18, 21, 28–30). In terms of protective factors against psychological distress, the need to inform children, who are constantly exposed to news about the epidemic, about the changes caused by the situation they are facing, clarifying any doubts to mitigate possible reactions, such as anxiety and panic, has been reported (31, 32). Other valuable strategies consist of multimedia entertainment and reading, a balanced diet and exercise, regular sleep patterns and good personal hygiene, and maintaining a daily routine (21, 29). Other valuable strategies consist of multimedia entertainment and reading, a balanced diet and exercise, regular sleep patterns and good personal hygiene, and maintaining a daily routine (21, 29). During confinement, parents should take confinement as an opportunity to engage their children through joint activities to strengthen family bonds and improve their autonomy (24).

As we have seen, during the COVID-19 era, in addition to a decrease in bacterial and viral infections due to home isolation, flu vaccination, mask use, and handwashing (33–35), was an increase in environmental stressors that produced adverse effects in parents and their children with both typical development and neurodevelopmental disorders (36). Also, for PANS/PANDAS to prepare therapeutic interventions, we should consider these environmental factors to produce the flare-up of symptoms.

## Aims

Starting from the diagnostic criteria and the symptomatic course that characterizes patients with PANDAS/PANS, in our exploratory study, we wanted to investigate the impact of the block on the clinical-psychological course of the syndrome (increase, stabilization, or reduction of symptoms). We hypothesized that reduced exposure to viral and bacterial agents that cause flare-ups of PANDAS/PANS symptoms could favor a reduction in clinical symptoms characterized by tics, doc, anxiety, irritability, eating problems, or other secondary symptoms (7). Thus, the first objective was to assess the course of symptoms during lockdown by asking parents to indicate whether they had observed a change in the frequency and intensity of symptoms and whether they had noticed the onset of new related symptoms.

Second, the study aimed to detect protective factors (environmental, social, family, and educational) for the management of symptoms present before and during the lockdown (e.g., parenting strategies, physician monitoring and continuation of therapies, new routines, sports or recreational activities online, social contacts with the outside world). The hypothesis was that these factors could prevent symptom flare-ups.

The third was to investigate risk factors for symptom escalation or reduction. Thirdly, parents were asked to identify and describe risk factors for symptom escalation (e.g., socio-demographic characteristics, social isolation, suspension of school, sports, recreational and therapy activities, family stress, loss of routine and support figures, or fears related to Covid). Finally, the aim was to detect whether environmental factors could generate a compromise or the onset of new symptoms even without bacterial infections.

## Methods

The multicenter study was based on completing an online proxy report questionnaire by parents of patients with PANS/PANDAS. The questionnaire can be accessed through a link created on the Google platform sent to families by the recruitment centers: Department of Pediatric Neurology - Policlinico Umberto I - Sapienza University of Rome, in collaboration with the Department of Clinical and Experimental Medicine - Division of Pediatrics and Childhood Neuropsychiatry - University of Catania, Complex Operating Unit - ASST Crema) and Child Neuropsychiatry Operating Unit - University of Cagliari.

All subjects followed with PANDAS/PANS treatment by referral centers were included; some of the patients' parents were also members of the PANDAS Italia ODV Association, which contributed to sending the questionnaire. Parents who filled out the questionnaire were asked to consent to the processing of data in aggregate form. One hundred and eight parents of children, adolescents, and young adults aged 3–21 years (mean age = 10.11 years) participated in the study, including 25 females and 83 males. The inclusion criteria concerned the maximum age of patients indicated by the experts (6) and were followed up at the participating hospital centers with a diagnosis of PANDAS or PANS. Some of these patients were also supported by the PANDAS Italia ODV Association, which collaborated for the sent link. The survey is open during the block from April 14 to May 3, 2020.

## Materials

### Description of the Tool

For data collection, a semi-structured questionnaire was constructed consisting of 22 questions divided into four sections. It was decided to use mainly closed answers to select the information based on the hypotheses previously described.

The first section (Q.1-11) includes sociodemographic information: child's age, child's gender, region, marital status, first parent's age, first parent's school attendance, second parent's age, second parent's school attendance, number of children in the family, type of house, parents' working condition. This section is characterized by open (Q.1; 3; 11), dichotomous (Q.2), or multiple-choice (Q.4-11) questions. The responses were subsequently used with the dual purpose of describing the profile of the participants and, secondly, of detecting potential risk or protective factors associated with the increase or reduction of symptoms during the lockdown.

The second section (Q.12-16) includes information on the symptoms observed in children during the lockdown, grouped into two areas: emotional and cognitive (sadness/withdrawal/depression, behavioral and physiological (items 12; 13). The following questions concern (Q.14-16) the symptoms present before quarantine, their evolution during the lockdown (reduction, stabilization, increase), and finally, new symptoms. In this section, dichotomous (item 14), multiple choice (Q.12; 13; 15), or open (Q.16) questions were used in order to detect the presence of core PANDAS/PANS and other symptoms, but also their course and the possible onset of new symptoms related to environmental factors.

The third section (Q.17-20) includes parenting strategies or actions to contain the child's problematic behavior. Multiple choice questions describe relational, distracting, or educational strategies (Q.17). This question is followed by dichotomous questions (Yes, No) on perceived efficacy (item 18) and open questions on the best strategies found for symptom containment (Q.19) and on other strategies to recommend to parents (Q.20). The strategies were included in the questionnaire to measure the actions that obtain the best results with young people affected by the syndrome and establish which measures may constitute protective factors concerning acute symptomatic.

The fourth section (D.21; 22) includes multiple-choice questions on other tools and actions that, according to the parents, can promote remission (Q.21) or elevation of symptoms (Q.22). Both questions also include an open space in which further suggestions can be entered. Thanks to these questions, it was possible to collect information on the potential effects of environmental variables on the development/course of symptoms and parents' perception about the actions they can activate or avoid supporting their children (Table 1).

**Table 1:** Structure and contents of the questionnaire: sections - information on the questions - number of questions.

**Sections**	**Informations**	**Questions *N***.
I- Sociodemographic	Children's parents, family, and logistics information.	1–11
II- Symptoms	Symptomatic characteristics before and during the block (type, course, or onset).	12–16
III- Parental strategies	Parental behaviors; effectiveness of parental behaviors; recommended strategies.	17–20
IV- Other tools/actions	Factors for symptom reduction; Factors for symptoms increased.	21–22

To proceed with the statistical analysis, and in line with the three initial hypotheses: (1) the condition of isolation reduces exposure to the prevention of exacerbation or the onset of new symptoms; (2) positive parenting strategies and specific environmental characteristics can be a protective factor against the recurrence of symptoms; (3) inadequate parenting, treatment or environmental characteristics, can constitute risk factors and increase symptoms, the following statistical analyzes were carried out: Section I (Sociodemographic): Description of the study sample through descriptive statistics and frequency analysis. For the answers, *n*. from 1 to 8 of this section, the items relating to the family composition (presence of 2 or more children; separated/divorced or single/widowed parents), the job position (unemployed/layoff), and the condition were considered potential risk factors. Housing (house without external space) and analyzed by comparing them with the variables “increase in symptoms” (Q.15 = yes) and “new symptoms” (Q.16 = yes). The articles from question *n*. 10 were grouped into two dichotomous variables (a–e = house with external space/f–j = house without external space).

Section II (Symptoms): descriptive analyzes and calculates the frequencies of all the items contained in questions *n*. 1-16. Comparison between symptoms presents during lockdown (Q.12 and 13) with their clinical course (Q. 14-16).

Section III (Strategies): in addition to the descriptive analysis and the frequencies of all the responses in this section, a subsequent comparison was made between items Q.17 (a, b, c) - Q.18 and the increased symptoms variables (=no) and new symptoms (=no), considering “positive” parenting strategies and perceived self-efficacy as potential protective factors. On the contrary, article *n*. 17.d, as compared with increased symptoms and the onset of new symptoms (=yes).

Section IV (Tools/action): after the descriptive and frequency analysis of all responses, Q.21 was compared with the decrease in symptoms and the lack of new symptoms (increased symptoms and new symptoms = no), while Q.22 was compared with increased symptoms and the onset of new ones.

### Statistical Analyses

Statistical analysis was conducted using SPSS 18.0 software for Windows (SPSS, Chicago, IL, USA). The Kolmogorov-Smirnov test was used to determine whether the variables were normally distributed. Normally distributed data are described as means ± standard deviations (SD). Group differences were analyzed with the Kruskal-Wallis test (for non-normally distributed data) or analysis of variance (ANOVA). Differences between percentages were assessed with the χ^2^-test. Bivariate analysis was performed with Spearman's correlation; a *p*-value < 0.05 was considered statistically significant. A binary logistic regression analysis was used to estimate the odds ratio and 95% IC of the variables associated with the increased symptoms during the block.

## Results

### Section I: Socio-Demographic Information

Through the analysis of the frequencies on demographic data, a sample of 108 PANDAS/PANDAS patients aged between 3 and 21 years (*M* = 10.7, SD = 3.21; Female: M = 9.99; Male: M = 10.11) (Table 2), was identified, 23% (*n* = 25) made up of females and 77% (*n* = 83) were males. The geographical distribution of the sample showed a prevalence of responses from Southern Italy 45.4% (*n* = 49), followed by the Center 31.5% (*n* = 34), from Northern Italy 22.2% (*n* = 24) and from abroad, 9% (*n* = 1). The information provided by the parents reported the following results: Family composition: 93% (*n* = 100) presence of two parents; 7% (*n* = 8) one parent only; 67% (*n* = 72) two children; 27% (*n* = 29) a child; 6% (*n* = 7) three or more children; 2). The age of the parent who completed the questionnaire places 73% (*n* = 79) of the sample in the 40–50 age range; 14% (*n* = 15) in the 30–40 years range; 13% (*n* = 14) in over 50 years with an upper secondary school level in 34% (*n* = 37), 32% degree (*n* = 35), 23% post-graduate (*n* = 25), middle school 8% (*n* = 9) and elementary school 2% (*n* = 2). Respondents to the questionnaire report an age of the partner between 40 and 50 years in 56% (*n* = 61) of the sample, over 50 years in 23% (*n* = 25), 30–40 years in 12% (*n* = 13) and under 30 years in 1% (*n* = 1) with high school diploma in 43% (*n* = 47), university degree 26% (*n* = 28), middle school 15% (*n* = 16), post-graduate 7% (*n* = 4) and elementary 1% (*n* = 1). This question was not answered by single parents. The occupational status of parents was distributed as follows: one parent in smartworking 52% (*n* = 56); both parents in smartworking 31% (*n* = 33); a parent unemployed or fired 10% (*n* = 11); both parents with work outside the home 6% (*n* = 7). Regarding the housing condition, several variables were explored to evaluate the possible effects on the symptomatological condition of children and young people. The variables were also divided into two macro-groups: house with outdoor space/ without external space presenting a higher frequency (65%) of dwelling with external space (Table 3).

**Table 2:** Differences between age and region means by gender.

**Gender**		**Age of children**
Female	Mean	9.96
	*N*	25
	SD	3.102
Male	Mean	10.11
	*N*	83
	SD	3.261
Total	Mean	10.07
	*N*	108
	SD	3.211

**Table 3:** Q.10 % of the type of house with/without outdoor space.

**Levels**	**Counts**	**% of total**	**Cumulative%**
House with outdoor space	26	24.1	24.1
Housing over 100 sqm (outdoor space)	24	22.2	46.3
Housing 70–100 sqm (outoor space)	17	15.7	62.0
Housing 50–70 sqm (outdoor space)	3	2.8	64.8
House without outdoor space	2	1.9	66.7
Housing over 100 sqm (without outdoor)	18	16.7	83.3
Housing 70–100 sqm (without outdoor)	12	11.1	94.4
Housing 50–70 sqm (without outdoor)	4	3.7	98.1
Housing up to 50 sqm (without outdoor)	2	1.9	100.0
Total	108	100.0	
House without outdoor space	38	35.2 %	35.2 %
House with outdoor space	70	64.8 %	100.0 %

### Section II: Evolution and Characteristics of Symptoms

**Figure 1:**
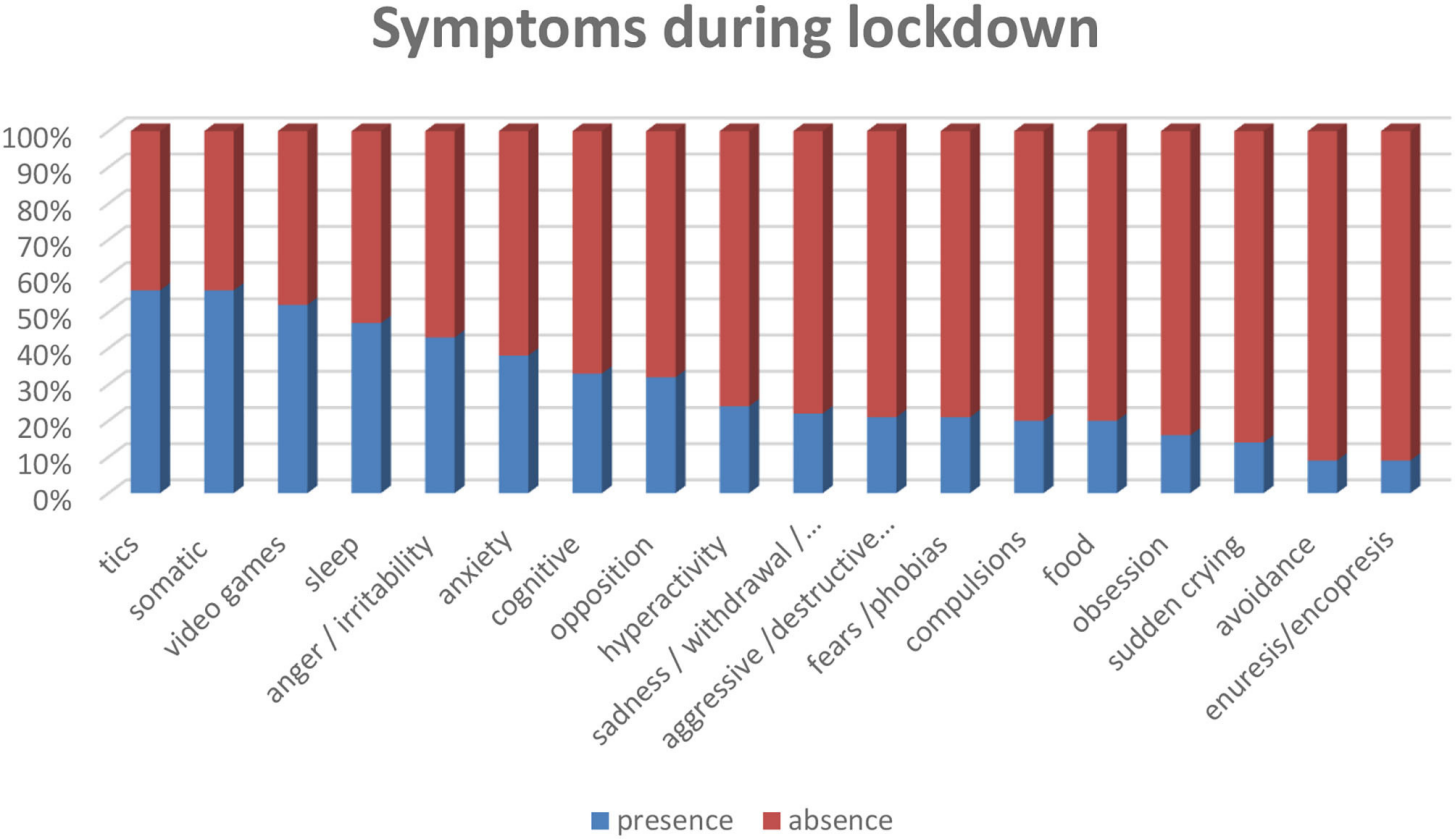
Frequencies of symptoms during the lockdown.

Among the symptoms during lockdown, the results show neurology disturbance like tics in 56% (*n* = 61), and cognitive difficulties (memory, attention, concentration) in 33% (*n* = 36) of the total sample. In the affective and thought areas, anger/irritability 43% (*n* = 46), anxiety 38% (*n* = 41), sadness/withdrawal/depression states in 22% (*n* = 24), sudden crying 14% (*n* = 15) and obsession 16% (*n* = 17). The most frequent behavioral problems concern the presence of somatic complaints 56% (*n* = 51), excessive use of video games 52% (*n* = 56), opposition 32% (*n* = 35), hyperactivity 24% (*n* = 26), compulsions 20% (*n* = 22), aggressive/destructive behavior and coprolalia 21% (*n* = 23), fears/phobias 21% (*n* = 23), avoidance 9% (*n* = 10). Finally, the physiological sphere presents sleep problems in 47% (*n* = 51) of the sample, food problems in 20% (*n* = 22) and episodes of enuresis or encopresis in 9% (*n* = 10). Frequencies and descriptions of symptoms divided by gender in the following (Figure 1 and Table 4).

**Table 4:** Descriptions of symptoms divided by gender.

**GENDER**		**CG**	**SD**	**IR**	**AN**	**SC**	**AG**	**OB**	**SO**	**TIC**	**OP**	**HY**	**CO**	**FP**	**VD**	**AV**	**EN**	**EA**	**SL**
Female	Mean	0.32	0.24	0.36	0.24	0.12	0.08	0.12	0.56	0.48	0.32	0.16	0.12	0.08	0.48	0.04	0.16	0.12	0.20
	*N*	25	25	25	25	25	25	25	25	25	25	25	25	25	25	25	25	25	25
	SD	0.476	0.436	0.490	0.436	0.332	0.277	0.332	0.507	0.510	0.476	0.374	0.332	0.277	0.510	0.200	0.374	0.332	0.408
Male	Mean	0.34	0.22	0.45	0.42	0.14	0.25	0.17	0.57	0.59	0.33	0.27	0.23	0.25	0.53	0.11	0.07	0.23	0.55
	*N*	83	83	83	83	83	83	83	83	83	83	83	83	83	83	83	83	83	83
	SD	0.476	0.415	0.500	0.497	0.354	0.437	0.377	0.499	0.495	0.471	0.444	0.423	0.437	0.502	0.313	0.261	0.423	0.500
Total	Mean	0.33	0.22	0.43	0.38	0.14	0.21	0.16	0.56	0.56	0.32	0.24	0.20	0.21	0.52	0.09	0.09	0.20	0.47
	*N*	108	108	108	108	108	108	108	108	108	108	108	108	108	108	108	108	108	108
	SD	0.474	0.418	0.497	0.488	0.347	0.411	0.366	0.498	0.498	0.470	0.430	0.405	0.411	0.502	0.291	0.291	0.405	0.502

*IN, increased symptoms; SL, sleep disturbance; EA, eating problems; EN, enuresis/encopresis; AV, avoidance; FP, fear/phobias; VD, video game addiction; CO, compulsions; HY, hyperactivity; OP, opposition; SO, somatic disorders; OB, obsessions; AG, aggression; SC, sudden crying; AN, anxiety; IR, irritability; SD, sadness/withdrawal/depression; CG, cognitive problems; NS, newsympotms*.

Compared to the core symptoms of PANDAS (6), the results show a high percentage of tics, 56%, while obsessions, compulsions, and eating disorders show percentages below 50%. Regarding the inclusion criteria of PANS (1), higher frequencies are found in sleep disturbances and irritability, which, however, do not exceed 50% of the frequencies.

The presence of tics characterizes the sample profile, and this data differentiates it from the average population, which, as described in the literature, report emotional symptoms such as anxiety and depression. However, it is difficult to differentiate the characteristics of PANS since, as is known, the clinical spectrum of the syndrome includes a heterogeneous constellation of secondary symptoms that are similar to those found in the average population during the lockdown. However, it is possible to note that as in the Gilles de la Tourette syndrome (35), the presence of tics prevails in our sample during the lockdown.

Psychometric analysis showed positive correlations in this sample between PANS/PANDAS symptoms (Table 5). The subgroup of symptoms that characterize Obsessive-Compulsive Disorder (OCD) shows significant correlations between obsessions and compulsions (*p* < 0.001), avoidance (*p* = 0.0020), and somatic complaints (*p* = 0.019). Obsessive thoughts are also connected with the use of video games (*p* = 0.027), and food problems (*p* = 0.020). There are associations between compulsion and sleep alterations (*p* = 0.027) and aggressivity (*p* = 0.012). The tics, which are the most frequent symptom in the sample, mostly occur independently, showing the correlation with avoidance behaviors (*p* = 0.002), cognitive problems (*p* = 0.009) and, a weak correlation with obsessions (*p* = 0.056); eating problems have a low frequency and are associated with sleep disturbances (*p* < 0.001), anxiety (*p* = 0.005) aggression (*p* = 0.012) and oppositional behaviors (*p* = 0.049).

**Table 5:** Correlation matrix: symptoms- symptoms increased- new symptoms.

	**CG**	**SD**	**IR**	**AN**	**SO**	**AG**	**OB**	**SC**	**TIC**	**OP**	**HY**	**CO**	**FP**	**VD**	**AV**	**EN**	**EA**	**SL**	**IN**
CG	—																		
SD	0.143	—																	
IR	0.133	0.025	—																
AN	0.579	0.005	0.313	—															
SO	0.559	0.002	0.043	0.459	—														
AG	0.008	0.028	< 0.001	0.406	0.009	—													
OB	0.194	0.442	0.143	0.405	0.785	0.127	—												
SC	0.497	0.038	0.117	0.019	0.396	0.350	0.019	—											
TIC	0.009	0.099	0.994	0.260	0.793	0.159	0.056	0.341	—										
OP	0.060	0.113	< 0.001	0.590	0.063	< 0.001	0.163	0.184	0.255	—									
HY	0.269	0.082	0.007	0.604	0.802	< 0.001	0.242	0.555	0.449	0.028	—								
CO	0.403	0.229	0.081	0.422	0.970	0.012	< 0.001	0.086	0.497	0.948	0.697	—							
FP	0.511	0.104	0.046	0.010	0.009	0.076	0.378	0.058	0.997	0.786	0.801	0.054	—						
VD	0.009	0.839	0.955	0.919	0.327	0.617	0.027	0.809	0.162	0.729	0.268	0.451	0.973	—					
AV	0.010	0.539	0.863	0.415	0.186	0.917	0.002	0.370	0.002	0.594	0.279	0.108	0.485	0.592	—				
EN	0.245	0.027	0.067	0.223	0.562	0.132	0.701	0.816	0.077	0.051	0.045	0.108	0.364	0.232	0.933	—			
EA	< 0.001	0.075	0.081	0.005	0.519	0.012	0.020	0.784	0.839	0.049	0.346	0.372	0.180	0.451	0.432	0.397	—		
SL	0.004	0.008	0.379	< 0.001	0.106	0.052	0.988	0.105	0.646	0.156	0.442	0.027	0.004	0.553	0.855	0.400	< 0.001	—	
IN	0.552	0.012	< 0.001	0.037	0.042	0.062	0.094	0.018	0.290	0.006	0.224	0.225	0.180	0.382	0.925	0.173	0.225	0.004	—
NS	0.100	< 0.001	0.104	0.160	0.302	0.216	0.945	0.137	0.054	0.379	0.449	0.721	0.216	0.194	0.412	0.120	0.052	0.022	0.026

Pearson's Correlation is significant at the *p < 0.05 level (2-tailed)—**p < 0.01 level(2-tailed).

*Abbreviations explained in the Table 4*.

Regarding the emotional area, most individuals have somatic complaints associated with obsessive symptoms but also with sadness/withdrawal/depression (*p* = 0.038); irritability is also very present in children and young people and closely related to aggressive and oppositional behaviors (*p* < 0.001), with hyperactivity (*p* = 0.007), crying (*p* = 0.043) and fear (*p* = 0.046). There are associations of depressed mood with crying (*p* = 0.02), irritability (*p* = 0.025), anxiety (*p* = 0.05), aggressive behavior (*p* = 0.028) and disturbances. somatic (*p* = 0.038). Children with depressed mood also have sleep disturbances (*p* = 0.008) and episodes of enuresis or encopresis (*p* = 0.027). Anxiety is mainly associated with the presence of fear or phobias (*p* = 0.010) and physiological alterations in sleep (*p* < 0.001) and nutrition (*p* = 0.005). The behavioral dimension presents a frequent excessive use of videogames associated with cognitive problems (*p* = 0.009) and the presence of obsessive thoughts (*p* = 0.027); of aggression in a strong relationship with opposition and hyperactivity (*p* < 0.001), with cognitive problems (*p* = 0.008), sudden crying (*p* = 0.009), as well as compulsions and eating disorders (as shown by the data previously described). Our sample also shows a strong link between oppositional behaviors and hyperactivity (*p* = 0.028), even if these manifestations appear less frequently, and both are associated with episodes of enuresis or encompassed (*p* = 0.045; *p* = 0.049). The opposition also has a relationship with eating problems, as discussed above. Sudden crying, which, as shown, is often present in children with irritability and aggression, is also present in association with feelings of fear or phobias (*p* = 0.009). At the same time, avoidance behaviors toward specific objects or situations occur in association with obsessions and tics. The presence of cognitive difficulties (memory, attention and concentration) is strongly correlated with aggression (*p* = 0.008), tics (*p* = 0.009), high use of video games (*p* = 0.009) and eating problems *p* < 0.001).

The results show statistically significant correlations between increased symptoms during lockdown and mood problems characterized by irritability (*p* < 001), sadness/depression (*p* = 0.012), somatic complaints (*p* = 0.18), anxiety (*p* = 0.37), and crying (*p* = 0.042). There is also an increase in oppositional behaviors (*p* = 0.006) and sleep problems (*p* = 0.004). An onset of new symptoms emerged in children and adolescents with depressed mood (*p* < 0.001) and eating problems (*p* = 0.022) and in general, with the group reporting an increase in symptoms during lockdown (*p* = 0.026).

Parents report symptoms in the period preceding lockdown in 84% (*n* = 91) of children associated with Covid emergency and an increase during quarantine in 71% (*n* = 77). The remaining 22% (*n* = 24) of the sample showed no difference in intensity and frequency of symptoms; only 7% (*n* = 7) reported a reduction in symptoms.

A small parents group of parents, 29% (*n* = 31), reported the onset of new symptoms in their children during the block, like more complex tics, negative emotions (sadness), generalized anxiety, irritability, and fears (of death, of the dark illness and leaving home) some of which were already present in other children of the sample, as shown in Table 6.

**Table 6:** Q16 new symptom.

**Levels**	**Counts**	**% of Total**	**Cumulative %**
Fear of virus	1	3.1	3.1
Generalized anxiety	3	9.4	12.5
Generalized fear	1	3.1	15.6
Complex motor tics	5	15.6	31.3
Compulsions	1	3.1	34.4
Fear of leaving home	2	6.3	40.6
Fear of virus	1	3.1	43.8
Sadness	3	9.4	53.1
Separation anxiety	1	3.1	56.3
Washing compulsions	1	3.1	59.4
Difficulty sleeping	2	6.3	65.6
Increased of videogames	1	3.1	68.8
Vocal tic	1	3.1	71.9
Sudden cry	1	3.1	75.0
Fear of disease	1	3.1	78.1
Fear of the dark	1	3.1	81.3
Fatigue	1	3.1	84.4
Oppositivity	1	3.1	87.5
Fear of death	1	3.1	90.6
Irritability	3	9.4	100.0

### Section III: Parental Strategies

**Figure 2:**
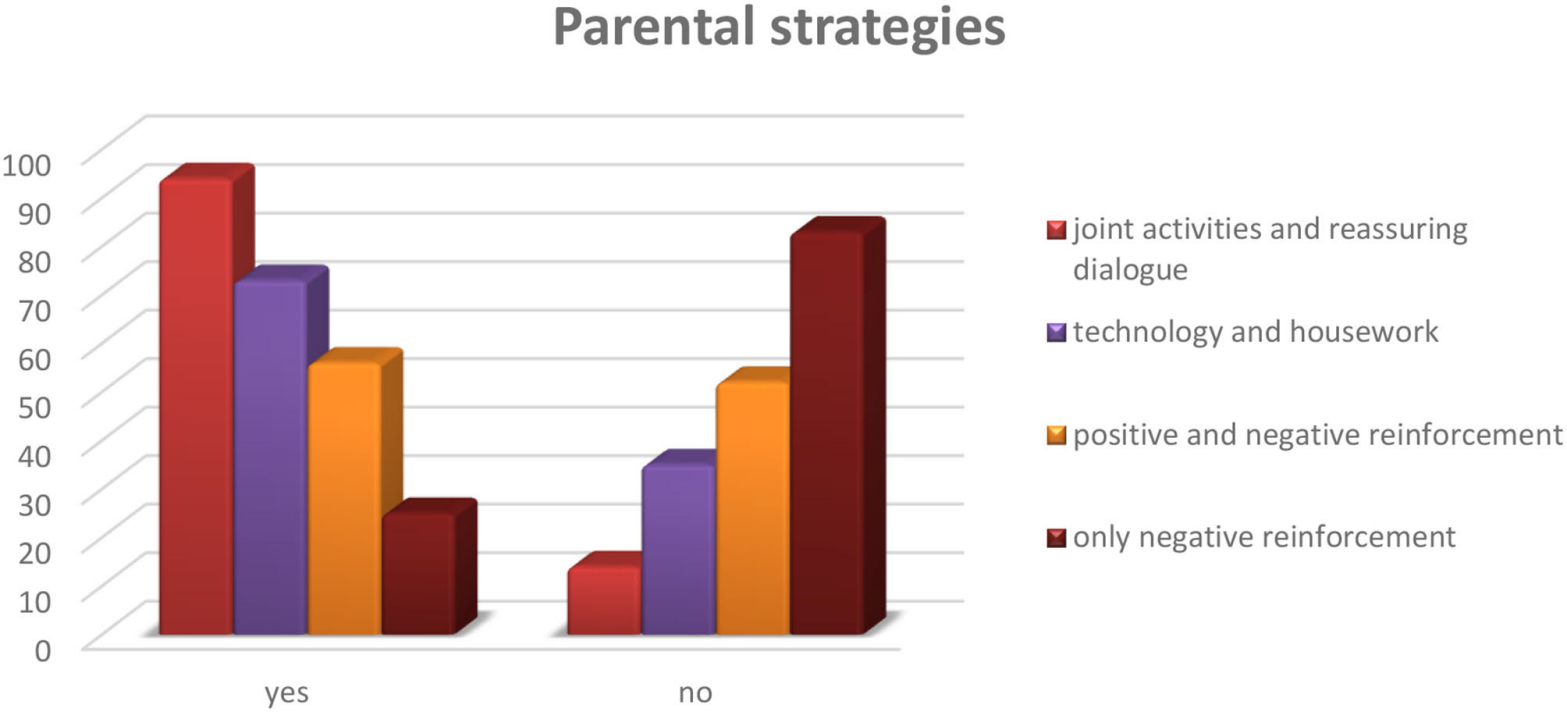
Q.17: What do you do to contain your child's discomfort?

Among the strategies implemented by parents to contain the stress of their children during the period of social isolation, 87% (*n* = 94) of parents reported greater use of relational activities: joint activities, reassurance, dialogue, and active listening. Sixty-eight percentage (*n* = 73) of parents used distraction-oriented strategies when their children were most stressed: use of technological tools and chores around the house. Fewer parents, 52% (*n* = 56), used psychoeducational methods through positive reinforcement when the child engaged in socially desirable behaviors and negative reinforcement when the child exhibited problematic behaviors (reward/punishment). Only a tiny percentage of caregivers, 23% (*n* = 25), used negative reinforcement, punishing the child in the presence of unwanted behavior. Seventy-one percentage (*n* = 77) of parents reported a positive efficacy of the strategies use (Figure 2).

The following are the strategies considered most effective by parents for containing acute episodes of their children. The most reported concern the relationship between caregiver and child/young maintained through dialogue or joint activities (Table 7).

**Table 7:** Q20 more positive strategies.

**Level**	**Counts**	**% of Total**	**Cumulative%**
Dialogue	24	31.6	31.6
Homework	8	10.5	42.1
Gratification	8	10.5	52.6
Joint activities	30	39.5	92.1
Manual activities	2	2.6	94.7
Physical contact	1	1.3	96.1
Sporting activities	2	2.6	98.7
Videogames	1	1.3	100.0

### Section IV: Other Tools/Actions

#### Factors for Symptom Reduction

**Figure 3:**
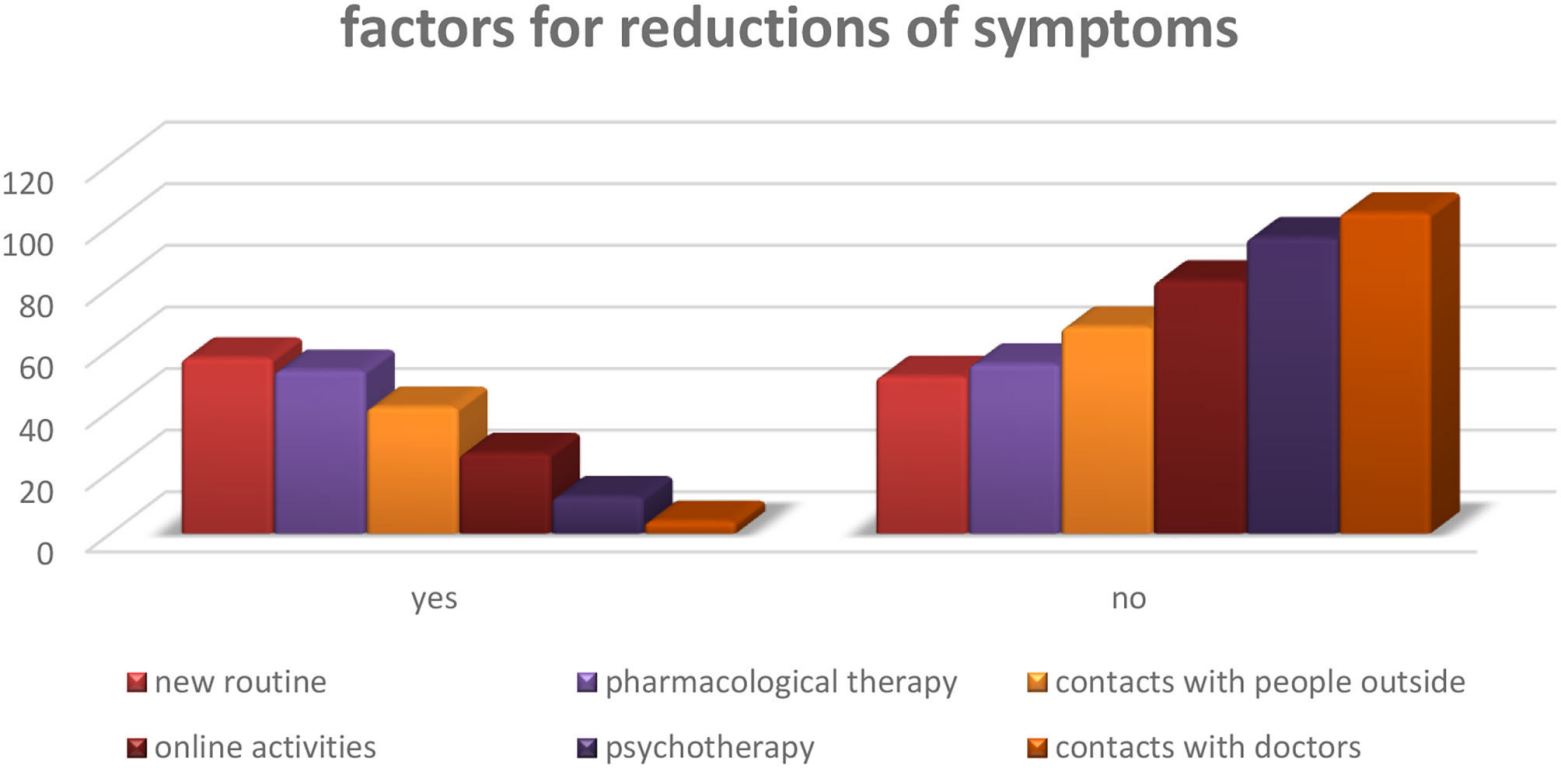
Q.21. Which factors do you think favor reduction of symptoms at this moment?

Factors perceived as effective by parents for prevention against increased symptoms include: establishing a new routine 53% (*n* = 57), continuing with drug therapy 50% (*n* = 53), maintaining contact with other people 38% (*n* = 41), online activities 24% (*n* = 26), online psychotherapy 11% (*n* = 12), followed by contact with doctors 4% (*n* = 4) (Figure 3).

The other factors indicated as predisposing the worsening of symptoms mainly concern distraction in the acute phases. However, it is essential to note that 79% of parents did not provide information in this regard (Table 8).

**Table 8:** Q21 other positive factors.

**Levels**	**Counts**	**% of Total**	**Cumulative %**
None	79	73.1	73.1
Videogames reduction	2	1.9	75.0
Distraction	8	7.4	82.4
Video-call	1	0.9	83.3
New routine	3	2.8	86.1
Unstructured routine	1	0.9	87.0
Have fun	1	0.9	88.0
Balanced diet	2	1.9	89.8
Ignore problematic behavior	1	0.9	90.7
Ignore its rituals	1	0.9	91.7
Problem solving with child	1	0.9	92.6
Reward when nervous	1	0.9	93.5
Empathy	2	1.9	95.4
Joint activities	3	2.8	98.1
Respect rules	1	0.9	99.1
Physical contact	1	0.9	100.0

#### Factors for Symptoms Increased

**Figure 4:**
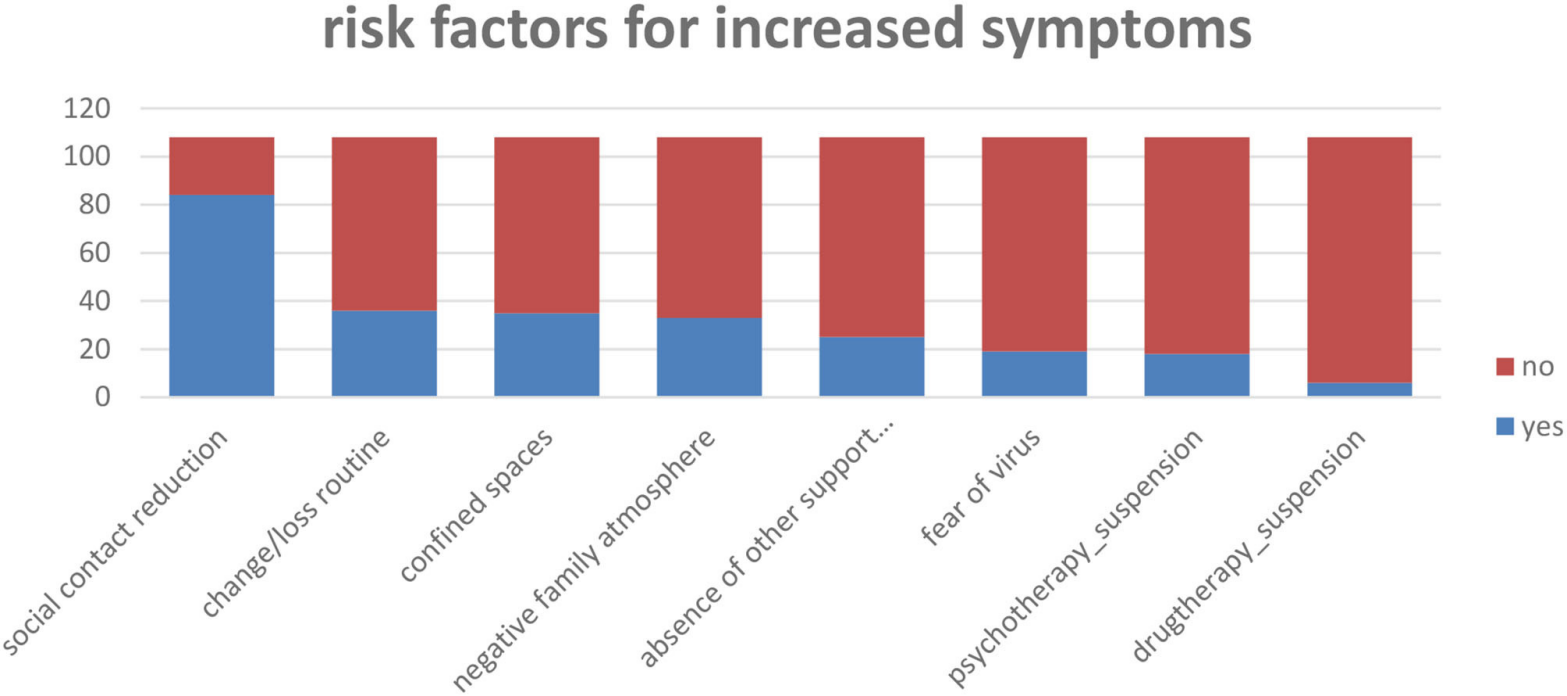
Q.22. What factors do you think interfere negatively with symptom control in this period?

As regards the risk factors perceived by parents for the increase in discomfort in children, the reduction of social contacts emerges more frequently, which also includes the suspension of extracurricular activities 78% (*n* = 84), followed by change or loss routine 33% (*n* = 36), restricted living space 32% (*n* = 35), followed by negative family climate 30% (*n* = 32), absence of support figures 23% (*n* = 25), the fear of contracting the virus on the part of parents or of the child 18% (*n* = 19), 17% (*n* = 18), and of psychotherapy 17% (*n* = 18), or pharmacotherapy 6% (*n* = 6) (Figure 4).

A very low percentage of parents also provided open answers on the factors they believe contribute to the increased risk of exacerbating their children's symptoms (Table 9).

**Table 9:** Q22 other negative factors.

**Levels**	**Counts**	**% of Total**	**Cumulative %**
None	102	94.4	94.4
Febrile episodes	1	0.9	95.4
Parental stress	1	0.9	96.3
Another child with Pandas	1	0.9	97.2
Conflict with teacher	1	0.9	98.1
Hypertechnology use	1	0.9	99.1
Discontinued reahabilitation therapy	1	0.9	100.0

### Risk and Protection Factors (Tables 11-15)

As previously described, a further statistical analysis was conducted through binomial logistic regressions to determine the risk factors associated with the increase in symptoms and the protective factors associated with the reduction/stability of symptoms during the lockdown. Regarding sociodemographic factors (Section I), the analysis did not provide significant values. This result can mean that determinants related to gender, age, family composition, housing, and working conditions do not predict the increase or the onset of new symptoms in the sample analyzed.

We then applied the same statistical analysis for the second section (symptoms), paying particular attention to the primary symptoms that characterize PANDAS (obsession, compulsion, eating disorder, and tic). However, these symptoms were not significant in explaining the increase. It is also important to note that symptoms before the block are not a predictor for the general increase (*p* = 0.145). In comparison, those representing a statistically significant predictor for the increase are irritable mood (*p* = 0.002) and oppositional behavior (*p* = 0.56). In conclusion, the presence of symptoms before lockdown, including the four main symptoms of the syndrome, does not justify a prediction of increased symptoms in this group over time (Table 10).

**Table 10:** Risk and Protective Factors for the Course of Symtomps.

	**Protection factors**	**Risk factors**
Sociodemographic		
Symptoms		Irritability
		Oppositivity
Strategies	Self-efficacy	
Other factors		Fear of contracting virus

Finally, the analysis of parental strategies and other tools or actions used by parents to contain the discomfort of children and young people (Sections III and IV) shows that the perception of parental self-efficacy referred to the strategies used with children can determine over time a containment of symptoms (effectiveness: *p* = 0.027) (Tables 11, 12). While the fear of contracting the virus is the factor that may have favored the increase or the onset of new symptoms (increase symptoms *p* = 0.028; Onset of new symptoms *p* = 0.002) (**Tables 14**, **15**).

**Table 11:** Regression between section II and symproms_increased.

	***B***	**E.S**.	**Wald**	**df**	**Sig**.	**Exp (*B*)**
SD	0.935	1.068	0.767	1	0.381	2.548
IR	2.628	0.869	9.141	1	0.002	13.849
AN	1.295	0.775	2.791	1	0.095	3.652
SC	2.176	1.517	2.058	1	0.151	8.814
AG	0.814	1.246	0.427	1	0.513	2.258
OB	1.963	1.256	2.443	1	0.118	7.120
SO	1.076	0.697	2.383	1	0.123	2.932
TIC	−0.054	0.687	0.006	1	0.938	0.948
OP	1.669	0.874	3.647	1	0.056	5.306
HY	−1.090	0.873	1.560	1	0.212	0.336
CO	−0.375	1.008	0.138	1	0.710	0.687
FP	−1.212	0.964	1.582	1	0.208	0.297
VD	0.681	0.619	1.210	1	0.271	1.975
AV	−0.434	1.344	0.104	1	0.747	0.648
EN	1.533	1.447	1.123	1	0.289	4.630
EA	−0.486	1.019	0.227	1	0.633	0.615
SL	1.933	0.761	6.455	1	0.011	6.909
PPS	−1.323	0.908	2.125	1	0.145	0.266

*Binomial logistic regression is significant at the p < 0.050*.

*Abbreviations explained in Table 4; abbreviation added: PPS, previous presence of symptoms*.

**Table 12:** Regression between positive factors and not onset of new symptoms (section III-IV).

	***B***	**E.S**.	**Wald**	**df**	**Sig**.	**Exp (*B*)**
Relation strategies (joint activities and reassuring) (Dialogue)	−0.735	0.866	0.721	1	0.396	0.479
Distraction (technology and housework)	0.430	0.610	0.498	1	0.481	1.538
Psychoeducational (positive and negative reinforcement)	−0.507	0.566	0.804	1	0.370	0.602
Pharmacological therapy	−0.232	0.304	0.584	1	0.445	0.793
Psychotherapy	−1.434	1.050	1.864	1	0.172	0.238
Contacts with doctors	−0.143	1.624	0.008	1	0.930	0.866
Contacts with people outside	−0.010	0.575	0.000	1	0.986	0.990
Online activities	−1.083	0.700	2.395	1	0.122	0.339
New routine	0.130	0.545	0.057	1	0.812	1.139

*Binomial logistic regression is significant at the p < 0.050*.

The chart below summarizes the potential risk and protective factors that have shown significant associations with increasing or decreasing symptom stability during the block (Table 10).

## Discussion

The literature has reported that during the COVID19 pandemic, the mental health status of children and adolescents with and without neurodevelopmental or neuropsychiatric disorders changed completely. During isolation, children and adolescents developed greater vulnerability due to social isolation and other environmental factors reassurance (20–23). In more severe cases, there may be elevated symptoms in the sphere of mood disorders (anxiety and depression), sleep and eating disorders, and the presence of obsessive-compulsive (wash) traits in clinical populations (24, 26). Among the possible triggers of psychological distress in children and adolescents, recent studies report parental stress and change in daily routines. Given the relapsing characteristics of PANS/PANDAS caused by viral and bacterial triggers, our exploratory study aimed to assess the course of symptoms during the blockade, hypothesizing psychopathological stabilization or reduction due to reduced exposure to infectious agents. Contrary to our hypotheses, the study results on 108 children and youths aged 3–21 years (*M* = 10.7, SD = 3.21) with PANS/PANDAS syndrome show an increase in symptoms during the lockdown in 71% of the sample. Psychometric analyses allowed us to exclude a relationship between core PANDAS symptoms (obsessions, compulsions, eating disorders, and tics) and symptom increase or the presence of symptoms before lockdown and their increase over time. The increase in symptoms is best explained by the presence of sleep disturbances and emotional lability such as sadness/depression, somatic complaints, anxiety, crying and irritability (Table 5). The increase during lockdown also appears to be related to the onset of new symptoms in children and adolescents with depressed mood and eating problems (Table 5). Furthermore, irritability and oppositionality are significant predictors of acute exacerbation (Tables 11–13). Also statistically significant in predicting the increase and onset of new symptoms is the factor linked to the effects of pandemic stress, such as the fear of contracting the virus (Tables 14, 15). It is possible to hypothesize that vulnerability to infections has amplified the fear that is generally already present in these young people and their caregivers. No significant associations for symptom reduction have been identified between parental strategies or other actions initiated by parents, but the study demonstrates that the effectiveness perceived by caregivers on the strategies used can reduce the risk of exacerbation.

**Table 13:** Regression between positive factors and not increased symptoms (section III-IV).

	***B***	**E.S**.	**Wald**	**df**	**Sig**.	**Exp (*B*)**
Relation strategies (joint activities and reassuring) (dialogue)	0.231	0.752	0.094	1	0.759	1.260
Distraction (technology and housework)	−0.821	0.565	2.116	1	0.146	0.440
Psychoeducational (positive and negative reinforcement)	0.463	0.476	0.945	1	0.331	1.589
Effectiveness	1.207	0.546	4.880	1	0.027	3.343
Pharmacological therapy	−0.020	0.240	0.007	1	0.933	0.980
Psychotherapy	1.272	0.909	1.958	1	0.162	3.568
Contacts with doctors	−0.246	1.323	0.035	1	0.852	0.782
Contacts with people outside	−0.426	0.500	0.725	1	0.395	0.653
Online activities	0.785	0.627	1.567	1	0.211	2.192
New routine	−0.333	0.479	0.484	1	0.486	0.716

*Binomial logistic regression is significant at the p < 0.050*.

**Table 14:** Regression between negative factors and increased symptoms (section IV).

	***B***	**E.S**.	**Wald**	**df**	**Sig**.	**Exp (*B*)**
Psychoeducational only negative reinforcement	1.044	0.656	2.529	1	0.112	2.840
Family climate negative	1.243	0.632	3.870	1	0.049	3.465
Confined spaces	0.360	0.552	0.425	1	0.514	1.433
Change or loss routine	1.065	0.608	3.071	1	0.080	2.900
Absence of other support	0.768	0.743	1.071	1	0.301	2.156
Fear of contracting virus	2.365	1.079	4.798	1	0.028	10.640
Reduction social contact	0.183	0.626	0.085	1	0.770	1.201
Psychotherapy suspension	0.647	0.768	0.710	1	0.400	1.909
Drugtherapy suspension	0.058	1.393	0.002	1	0.967	1.060
Previous presence	−0.165	0.735	0.050	1	0.823	0.848

*Binomial logistic regression is significant at the p < 0.050*.

**Table 15:** Regression between negative factors and onset of new symptoms (section III-IV).

	***B***	**E.S**.	**Wald**	**df**	**Sig**.	**Exp (*B*)**
Psychoeducational only negative reinforcement	0.323	0.556	0.338	1	0.561	1.381
Family climate negative	0.403	0.523	0.593	1	0.441	1.496
Confined spaces	0.389	0.494	0.621	1	0.431	1.476
Change or loss routine	−0.526	0.542	0.942	1	0.332	0.591
Absence of other support	0.443	0.590	0.563	1	0.453	1.557
Fear of contracting virus	1.762	0.577	9.335	1	0.002	5.826
Reduction social contact	0.314	0.631	0.248	1	0.618	1.369
Psychotherapy suspension	0.928	0.643	2.085	1	0.149	2.530
Drugtherapy suspension	−0.606	1.202	0.254	1	0.614	0.546
Previous presence	−0.720	0.602	1.431	1	0.232	0.487

*Binomial logistic regression is significant at the p < 0.050*.

## Conclusion

This preliminary study highlights the importance of studying and investigating the causes of increased symptoms in children with PANDAS/PANS. As described, stressful events can contribute to the worsening of the disease and generate new symptoms. In particular, environmental, family, and social changes in the course of clinical symptoms in PANDAS/PANS patients should be investigated. The importance of managing emotions and problematic behaviors is also highlighted. It has also been found that parents' perception of self-efficacy concerning their educational and caring skills can act against the increase in symptoms. This information deduced from the study results is consistent with the guidelines on the clinical management of the syndrome, which favor a combined approach between pharmacotherapy, psychotherapy, and parent training (1).

## Limits

An experimental proxy-report questionnaire not yet standardized and validated on the PANS/PANDAS pediatric clinical sample was used for the exploratory study. To date, there are no specific standardized tools for PANS and PANDAS syndrome.

There is also a small sample size (*N* = 108) and the absence of a control group (pre-lockdown or children without PANDAS/PANS). Moreover, the number of patients is not fully representative of the Italian population (mainly Central and Southern Italy). It would be interesting to evaluate the exact long-term dimensions to see the course of symptoms after covid. It would also be helpful to conduct a new study focusing on the impact of stressful events on the clinical course of the syndrome.

## Data Availability Statement

The raw data supporting the conclusions of this article will be made available by the authors, without undue reservation.

## Ethics Statement

Ethical review and approval was not required for the study on human participants in accordance with the local legislation and institutional requirements. Written informed consent to participate in this study was provided by the participants' legal guardian/next of kin.

## Author Contributions

CG: wrote the paper design study. AS: design study. LL: validated. AZ: edit. SS, AG, and PP: data summary. GB: statistics. AB and GG: collaboration. All authors contributed to the article and approved the submitted version.

## Conflict of Interest

The authors declare that the research was conducted in the absence of any commercial or financial relationships that could be construed as a potential conflict of interest.

## Publisher's Note

All claims expressed in this article are solely those of the authors and do not necessarily represent those of their affiliated organizations, or those of the publisher, the editors and the reviewers. Any product that may be evaluated in this article, or claim that may be made by its manufacturer, is not guaranteed or endorsed by the publisher.

## References

[1] Thienemann M Murphy T Leckman J Shaw R Williams K Kapphahn C. Clinical management of pediatric acute-onset neuropsychiatric syndrome: part I - psychiatric and behavioral interventions. J Child Adolesc Psychopharmacol. (2017) 566–73. 10.1089/cap.2016.0145 28722481 PMC5610394

[2] Allen AJ Leonard HL Swedo SE. Case study: a new infection-triggered, autoimmune subtype of pediatric OCD and Tourette's syndrome. J Am Acad Child Adolesc Psychiatry. (1995) 34:307–11. 10.1097/00004583-199503000-00015 7896671

[3] Swedo Susan E Rapoport JL Leonard H Lenane M Cheslow D. Obsessive-compulsive disorder in children and adolescents: clinical phenomenology of 70 consecutive cases. Arch Gen Psychiatry. (1989) 46:335–41. 10.1001/archpsyc.1989.01810040041007 2930330

[4] Swedo SE Leonard HL Garvey MA Mittleman BB. PANDAS: pediatric autoimmune neuropsychiatric disorders associated with strep - is this a new species of childhood-onset obsessive-compulsive disorder and tourette's syndrome? Eur Neuropsychopharmacol. (1996) 155:264–71. 10.1016/0924-977X(96)83025-2

[5] Swedo SE Leonard HL Garvey M Mittleman B Allen AJ Perlmutter S. Pediatric autoimmune neuropsychiatric disorders associated with streptococcal infections: clinical description of the first 50 cases. Am J Psychiatry. (1998) 155:264–71. 9464208 10.1176/ajp.155.2.264

[6] Swedo SE. From research subgroup to clinical syndrome: modifying the PANDAS criteria to describe PANS (pediatric acute-onset neuropsychiatric syndrome). Pediatr Therap. (2012) 2:113. 10.4172/2161-0665.1000113

[7] Chang K Frankovich J Cooperstock M Cunningham MW Latimer ME Murphy TK. Clinical evaluation of youth with pediatric acute-onset neuropsychiatric syndrome (PANS): recommendations from the 2013 PANS consensus conference. J Child Adolesc Psychopharmacol. (2015) 25:3–13. 10.1089/cap.2014.0084 25325534 PMC4340805

[8] Pavone P Parano E Battaglia C Marino S Trifiletti RR Marino SD. Severe psychotic symptoms in youth with PANS/PANDAS: case-series. J Child Adolesc Psychopharmacol. (2020) 30:567–71. 10.1089/cap.2020.0050 32700992

[9] Gagliano A Galati C Ingrassia M Ciuffo M Alquino MA Tanca MG. Pediatric acute-onset neuropsychiatric syndrome: a data mining approach to a very specific constellation of clinical variables. J Child Adolesc Psychopharmacol. (2020) 30:495–511. 10.1089/cap.2019.0165 32460516

[10] Loffredo L Spalice A Salvatori F de Castro G Guido CA Zicari AM. Oxidative stress and gut-derived lipopolysaccharides in children affected by paediatric autoimmune neuropsychiatric disorders associated with streptococcal infections. BMC Pediatr. (2020) 20:127. 10.1186/s12887-020-02026-8 32188439 PMC7079429

[11] APA, American Journal of Psychiatry American Psychiatric Association. Diagnostic and Statistical Manual of Mental Disorders. 5th ed. APA (2013).

[12] Gori A Leone F Loffredo L Cinicola BL Brindisi G de Castro G. COVID-19-related anosmia: the olfactory pathway hypothesis and early intervention. Front Neurol. (2020) 11:956. 10.3389/fneur.2020.00956 33013637 PMC7511833

[13] Kabeerdoss J Pilania RK Karkhele R Kumar TS Danda D Singh S. Severe COVID-19, multisystem inflammatory syndrome in children, and Kawasaki disease: immunological mechanisms, clinical manifestations and management. Rheumatol Int. (2021) 41:19–32. 10.1007/s00296-020-04749-4 33219837 PMC7680080

[14] Parisi GF Brindisi G Indolfi C Diaferio L Marchese G Ghiglioni DG. Upper airway involvement in pediatric COVID-19. Pediatr Allergy Immunol. (2020) 31:85–8. 10.1111/pai.13356 PMC7753446 33236430

[15] Cucinotta D Vanelli M. WHO declares COVID-19 a pandemic. Acta Biomed. (2020) 91:157–60. 10.23750/abm.v91i1.9397 32191675 PMC7569573

[16] Guido CA Amedeo I Avenoso F Bruni J Zicari AM Loffredo L. Risk factors and mental health promotion strategies in children during COVID-19. Front Public Health. (2020) 8:580720. 10.3389/fpubh.2020.580720 33178664 PMC7593512

[17] Liu S Liu Y Liu Y. Somatic symptoms and concern regarding COVID-19 among Chinese college and primary school students: a cross-sectional survey. Psychiatry Res. (2020) 289:113070. 10.1016/j.psychres.2020.113070 32422501 PMC7227526

[18] Xie X Xue Q Zhou Y Zhu K Liu Q Zhang J. Mental health status among children in home confinement during the coronavirus disease 2019 outbreak in Hubei Province, China. In JAMA Pediatrics. (2020) 174:898–900. 10.1001/jamapediatrics.2020.1619 PMC7182958 32329784

[19] Racine N Cooke JE Eirich R Korczak DJ McArthur BA Madigan S. Child and adolescent mental illness during COVID-19: a rapid review. Psychiatry Res. (2020) 292:113307. 10.1016/j.psychres.2020.113307 32707216 PMC7363598

[20] Flaudias V Iceta S Zerhouni O Rodgers RF Billieux J Llorca PM. COVID-19 pandemic lockdown and problematic eating behaviors in a student population. J Behav Addict. (2020) 9:826–35. 10.1556/2006.2020.00053 32976112 PMC8943668

[21] Jiao WY Wang LN Liu J Fang SF Jiao FY Pettoello-Mantovani M. Behavioral and emotional disorders in children during the COVID-19 epidemic. J Pediatr. (2020) 221:264–6.e1. 10.1016/j.jpeds.2020.03.013 32248989 PMC7127630

[22] Loades ME Chatburn E Higson-Sweeney N Reynolds S Shafran R Brigden A. Rapid systematic review: the impact of social isolation and loneliness on the mental health of children and adolescents in the context of COVID-19. J Am Acad Child Adolesc Psychiatry. (2020) 59:1218–39.e3. 10.1016/j.jaac.2020.05.009 32504808 PMC7267797

[23] Liu JJ Bao Y Huang X Shi J Lu L. Mental health considerations for children quarantined because of COVID-19. Lancet Child Adolesc Health. (2020) 4:347–9. 10.1016/S2352-4642(20)30096-1 32224303 PMC7118598

[24] Tanir Y Karayagmurlu A Kaya I Kaynar TB Türkmen G. Exacerbation of obsessive compulsive disorder symptoms in children and adolescents during COVID-19 pandemic. Psychiatry Res. (2020) 293:113363. 10.1016/j.psychres.2020.113363 32798931 PMC7837048

[25] Robertson MM Eapen V Rizzo R Stern JS Hartmann A Müller-Vahl KR. Gilles de la tourette syndrome: advice in the times of COVID-19. F1000Research. (2020) 9:257. 10.12688/f1000research.23275.2 32411359 PMC7195896

[26] Conti E Sgandurra G de Nicola G Biagioni T Boldrini S Bonaventura E. Behavioural and emotional changes during covid-19 lockdown in an italian paediatric population with neurologic and psychiatric disorders. Brain Sci. (2020) 10:918. 10.3390/brainsci10120918 PMC7760933 33260987

[27] Cusinato M Iannattone S Spoto A Poli M Moretti C Gatta M. Stress, resilience, and well-being in Italian children and their parents during the COVID-19 pandemic. Int J Environ Res Public Health. (2020) 17:8297. 10.3390/ijerph17228297 PMC7696524 33182661

[28] Rosen Z Weinberger-Litman SL Rosenzweig C Rosmarin DH Muennig P Carmody ER. Anxiety and distress amongthe first community quarantined in the U.S due to COVID-19: psychological implications for the unfolding crisis. Psyarxiv. (2020) 1–18. Preprint. 10.31234/osf.io/7eq8c

[29] Zhou SJ Zhang LG Wang LL Guo ZC Wang JQ Chen JC. Prevalence and socio-demographic correlates of psychological health problems in Chinese adolescents during the outbreak of COVID-19. Eur Child Adolesc Psychiatry. (2020) 29:749–58. 10.1007/s00787-020-01541-4 32363492 PMC7196181

[30] Oosterhoff B Palmer CA Wilson J Shook N. Adolescents' motivations to engage in social distancing during the COVID-19 pandemic: associations with mental and social health. J Adolesc Health. (2020) 67:179–85. 10.1016/j.jadohealth.2020.05.004 32487491 PMC7205689

[31] Dalton L Rapa E Stein A. Protecting the psychological health of children through effective communication about COVID-19. The Lancet Child and Adolescent Health. (2020) 4:346–7. 10.1016/S2352-4642(20)30097-3 32243784 PMC7270522

[32] Wang G Zhang Y Zhao J Zhang J Jiang F. Mitigate the effects of home confinement on children during the COVID-19 outbreak. Lancet. (2020) 395:945–7. 10.1016/S0140-6736(20)30547-X PMC7124694 32145186

[33] Patel S Thompson MD Slaven JE Sanders DB Ren CL. Reduction of pulmonary exacerbations in young children with cystic fibrosis during the COVID-19 pandemic. Pediatr Pulmonol. (2021) 56:1271–3. 10.1002/ppul.25250 33434352 PMC8014497

[34] Younie S Mitchell C Bisson MJ Crosby S Kukona A Laird K. Improving young children's handwashing behaviour and understanding of germs: the impact of A Germ's Journey educational resources in schools and public spaces. PLoS ONE. (2020) 15:e0242134. 10.1371/journal.pone.0242134 33227004 PMC7682880

[35] Van Brusselen D Vlieghe E Schelstraete P de Meulder F Vandeputte C Garmyn K. Streptococcal pharyngitis in children: to treat or not to treat? Eur J Pediatr. (2014) 173:1275–83. 10.1007/s00431-014-2395-2 25113742

[36] Bentenuto A Mazzoni N Giannotti M Venuti P de Falco S. Psychological impact of Covid-19 pandemic in Italian families of children with neurodevelopmental disorders. Res Dev Disabil. (2021) 109:103840. 10.1016/j.ridd.2020.103840 33383468 PMC9186313

