# Supplementary material for: The Impact of the COVID-19 Epidemic During the Lockdown on Children With the Pediatric Acute-Onset Neuropsychiatric Syndrome (PANDAS/PANS): The Importance of Environmental Factors on Clinical Conditions
Source: Front Neurol. 2021 Aug 11;12:702356. doi: 10.3389/fneur.2021.702356 (PMC8385147; doi:10.3389/fneur.2021.702356)
Supplement: Supplementary file 1 [file Data_Sheet_1.docx]

**Appendix**

**Tools:**

**PANS/PANDAS SURVEY DURING COVID-19 LOCKDOWN (english version)**

| **SECTION I: SOCIO-DEMOGRAPHIC INFORMATION** |
| --- |
| **1. Child age** ________________________ |
| **2. Child gender**  F  M |
| **3. Region of residence** ________________________ |
| **4. Marital status of parents** |
| Married/Cohabiting |
| Separated divorced |
| Single/Unmarried/Widowed |
| Reconstituted family |
| **5. Parents' age** |
| 20-30 Years |
| 30-40 Years |
| 40-50 Years |
| 50+ Years |
| **6.. Parents' school attendance** |
| Elementary school |
| Middle school |
| Secondary school |
| Degree |
| Post-Laurea |
| **7. Age Of Parent 2:** biological parent or partner in recomposed family (fill in only if there are two caregivers) |
| 20-30 Years |
| 30-40 Years |
| 40-50 Years |
| 50+ Years |
| **8. School Attendance Parent 2 (**fill in only in the presence of two caregivers) |
| Elementary school |
| Middle school |
| Secondary school |
| Degree |
| Post-Laurea |
| **9. Number of children** (living with the child with Pandas/Pans) |
| 1 (Only Child) |
| 2 |
| 3/+ |
| **10. Type of house** (select 1 or more answers) |
| House with outdoor space |
| House without outdoor space |
| Housing up to 50 sqm |
| Housing from 50 sqm to 70 sqm |
| Housing from 70 sqm to 100 sqm |
| Housing over 100 sqm |
| **11. Current working condition** (select 1 or more answers) |
| Working in smart working (1 parent only) |
| Working in smart working (both parents) |
| Work outside the home (both parents) |
| Unemployed/casse integration (1 parent only) |
| Unemployed/integration case (both parents) |
| Other ________________________ |
|  |
| **SECTION II: INFORMATION ABOUT THE SYMPTOMS OBSERVED IN THE CHILD** |
| In answering the questions, please focus only on the quarantine period. (select 1 or more answers) |
| **12. What indicators of distress are present in your child during this period?** (select 1 or more answers) |
| Anxiety |
| Fear or specific phobias |
| Sadness |
| Anger/Irritability |
| Closure/depression |
| Hyperactivity |
| Difficulty in one or more cognitive functions (memory, concentration, attention, language) |
| Oppositionality/poor compliance with rules |
| Obsessions |
| Other ________________________ |
| **13. Indicate if the following behaviors are present** |
| Compulsions (Rituals) |
| Avoidance of objects, situations or people |
| Sudden crying |
| Enuresis |
| Encopresis |
| Damage to objects or aggression towards people |
| Coprolalia |
| Sleep alteration (increased/reduced/difficulty falling asleep/ awakening at night) |
| Eating problems (hypo/hyperphagia/selective eating) |
| Tic |
| Increased use of video games or other technological tools |
| Excessive attention requirement (e.g., sleeping together, interrupting parent's activities) |
| Somatic complaints (headache, abdominal pain, nausea, vomiting, or other discomfort without organic causes) |
| **14. Were the symptoms/behaviors present before quarantine?**  Yes  No |
| **15. Have the symptoms/behaviors changed during the quarantine period**? |
| Have increased in intensity and/or frequency |
| Have decreased in intensity and/or frequency |
| Remained stable |
| **16. Has the children developed new symptoms/behaviors that you are concerned about related to the covid emergency?** |
| specify:________________________________________________________________________________ |
| **SECTION III: PARENT'S BEHAVIOR** |
| **17. What do you do to contain the child's/your child's distress?** (Select 1 or more answers) |
| Reassure him/her by talking to him/her |
| Try to physically contain him/her |
| Punish him/her (scolding/restricting or depriving him/her of games or other sources of pleasure) |
| Gratify him/her (comply with his/her requests, add privileges or compliment him/her for positive behavior) |
| Give more time to joint activities (e.g., playing, cooking, reading together, etc.). |
| Humor him/her by participating in his/her rituals. |
| Involve him/her in household activities (e.g. tidying up the room, setting the table, etc.). |
| Alter gratitude to punishment (in case of positive or negative behavior) |
| Allow him/her to spend a lot of time in front of the tv, tablet, pc or other |
| **18. Are the strategies used effective?**  Yes  No |
| **19. Which of the strategies indicated give the most positive results**? ( |
| answer if more than one is indicated)_______________________________________________________­­­­_­­­ |
| **20. Are there any other strategies you are using that you would recommend to parents?** |
| Specify:________________________________________________________________________________ |
|  |
| **SECTION IV: OTHER TOOLS/ACTIONS** (in this section we ask you to indicate which of the following tools or actions are helping to manage the issues highlighted in the previous sections) |
| **21. Which factors do you think favour reduction of symptoms at this moment (select 1 or more answers)** |
| Antibiotic therapy (including prophylaxis) |
| Psychopharmaceutics |
| Psychotherapy/Psychological support (only if ongoing) |
| Anti-inflammatories |
| Establish a new routine with activities that engage him/her daily |
| Participate in online activities (e.g., courses, workshops, etc.) |
| Maintain contact with the outside world (via smartphone, social networks, interactive games or other) |
| Increasing the frequency of telephone contact with the child's physicians |
| Other___________________________________________________________________________ |
| **22. What factors do you think interfere negatively with symptom control in this period?** |
| Family climate (e.g. conflict between parents or children, tension due to loss of job, etc.) |
| Confined spaces |
| Change or loss of family routine (e.g., schedules, habits, presence of both parents) |
| Absence of other supporting persons (e.g., grandparents, babysitter, etc.) |
| Fear in parents of contracting the virus |
| Child's fear of contracting the virus |
| Negative humor in the parents (e.g., excessive worry and/or sadness and/or fear and/or anger) |
| Isolation/reduction of social contacts of the child |
| Interruption of out of school activities (sports, afternoon courses of music, english or other) |
| Suspension of psychotherapy/psychological support |
| Suspension of Intramuscular antibiotic therapy |
| Other___________________________________________________________________________ |
